# Gender awareness among medical students in a Swiss University

**DOI:** 10.1186/s12909-020-02037-0

**Published:** 2020-06-03

**Authors:** Ilire Rrustemi, Isabella Locatelli, Joëlle Schwarz, Toine Lagro-Janssen, Aude Fauvel, Carole Clair

**Affiliations:** 1 Faculty of Biology and Medicine, University of Lausanne, Lausanne, Switzerland; 2 Center for Primary Care and Public Health (Unisanté), Lausanne, Rue du Bugnon 44, CH-1011 Lausanne, Switzerland; 3 Dept. of Primary and Community Care, Radboud University Medical Center, Geert Grooteplein 21, 6525 EZ Nijmegen, Netherlands; 4 Institute of Humanities in Medicine, Lausanne University Hospital (CHUV), Lausanne, Avenue de Provence 82, 1007 Lausanne, Switzerland

**Keywords:** Gender, Medical education, Gender bias, Gender stereotypes

## Abstract

**Background:**

Gender is an important social determinant, that influences healthcare. The lack of awareness on how gender influences health might lead to gender bias and can contribute to substandard patient care. Our objectives were to assess gender sensitivity and the presence of gender stereotypes among swiss medical students.

**Methods:**

A validated scale (N-GAMS – Nijmegen Gender Awareness in Medicine Scale), with 3 subscores assessing gender sensitivity (GS) and gender stereotypes toward patients (GRIP) and doctors (GRID) (ranging from 1 to 5), was translated into French and was distributed to all medical students registered at the University of Lausanne, Switzerland in April–May 2017. Reliability of the three subscales was assessed calculating the alpha Cronbach coefficient. Mean subscales were calculated for male and female students and compared using two sample t-tests. A linear model was built with each subscale as a dependent variable and students’ sex and age as covariables.

**Results:**

In total, 396 students answered the N-GAMS questionnaire, their mean age was 22 years old, 62.6% of them were women. GS and GRID sub-scores were not significantly different between female and male students (GS 3.62 for women, 3.70 for men, *p* = 0.27, GRID 2.10 for women, 2.13 for men, *p* = 0.76). A statistically significant difference was found in the GRIP subscale, with a mean score of 1.83 for women and 2.07 for men (*p* < 0.001), which suggests a more gender stereotyped opinion toward patients among male students. A trend was observed with age, gender sensibility increased (p < 0.001) and stereotypes decreased (GRIP *p* = 0.04, GRID *p* = 0.02) with students getting older.

**Conclusion:**

Medical students’ gender sensitivity seems to improve throughout the medical curriculum, and women students have less stereotypes towards patients than men do. The implementation of a gender-sensitive teaching in the medical curriculum could improve students’ knowledge, limit gender bias and improve patients’ care.

## Background

Gender is considered as a social determinant of health, at the same level as ethnicity and education. Social inequalities between men and women influence health at different levels from structural to individual health behaviors [1]. A strong call has been made in the last decades to systematically integrate sex and gender dimensions and to raise gender awareness in medical education, medical research and epidemiology [2, 3]. Gender awareness is the “ability to view society from the perspective of gender roles and how this has affected women’s needs in comparison to the needs of men” [4]. Thus, gender awareness aims toward better health for men and women. Lack of gender awareness leads to gender bias and can contribute to unfair patient care [5, 6]. There are two types of gender bias in medicine: *gender stereotype*, which is defined as the clinically unjustified difference of treatment between female and male patients; and *gender blindness*, which is defined by the inability to recognize differences when they are clinically pertinent [6, 7]. Gender stereotypes influence physician’s differential diagnosis and decisions of management. A common example of stereotypes is found in cardiovascular disease, where coronary heart disease is often underdiagnosed in women due to a different, biased management [8, 9]. Gender stereotypes are acquired in society through socialization of both men and women and are rooted in gendered roles, identities and representations. Gender blindness results from fundamental and clinical research that has been historically (and often still is) conducted predominantly on male participants, results being then extrapolated to women [10]. The common example is again in the treatments of cardiovascular diseases [10].

Gender stereotypes can be prevented through a gender-sensitive medical education [5]. In the European context, implementation of a gender perspective in medical education started mainly in 2002 in the Netherlands, with a successful research-project lead by Prof. Lagro-Janssen, applying the concept of *Gender mainstreaming*. In 4 years, gender and sex issues were implemented into the existing curriculum at all levels and specific lectures about gender awareness were launched in the medical school of Radboud University [11]. Integrating gender related lectures and implementing gender perspective in the specialties teaching showed results on preventing gender disparities in healthcare [8].

In Switzerland, despite the principle of equality between women and men being enshrined in the Federal Constitution since 1981, gender inequality is observed in many domains such as economic activity, salaries, share of domestic work, political representation [12]. For example, 65% of women manage all the domestic activities (OFS, 2013) in a heterosexual couple. In the professional domain, women have more part time jobs than men (59% had part time jobs in 2017) [13], and are paid less (in 2016, women are paid 19.6% less than men in the private sector) [14]. These divisions contribute to different health-related exposures and lifestyle behaviors, as well as to social stereotypes that are reflected also in the medical field and among medical staff.

In Switzerland, women entered the medical profession for the first time in 1867 [15], and today while the majority of medical students are women, they are still under represented in leading medical positions [16]. These gendered organizations of leadership might reinforce gender stereotypes in the clinical setting. In the University of Lausanne (UniL), gender studies were integrated in 2000 in the Faculty of Social and Political Sciences. From 2003, the UniL acknowledged the importance of gender in health, along with the Federal office of Public Health in Switzerland, which created a “Gender Health” service in 2001 [17]. The first lecture on gender and medicine was held to medical students in 2005 [17]. A platform of interdisciplinary gender studies (PLaGe – Plateforme en Etudes Genre) was created in 2012, aiming to gather all projects of the university around the question of gender, sexuality and sexual orientation [18].

Currently, UniL students follow a 2-h introduction to gendered medicine during their first year of studies, an optional 12 h seminar, and 2-h lectures on gender and health during the 4th and 5th year. To promote an integrated structured teaching of the gender dimension in medicine, the Faculty of Biology and Medicine at UniL is currently implementing a Gendered Medicine project. This project aims at integrating a gender dimension in all disciplines of pre-graduate medical education and apply gender regulated terms in research, such as including female participants or/and addressing the differences of sex and gender in the outcome of interest [19]. This study was conducted in the frame of this project, with the goal to assess gender awareness in medical students using a validated scale developed in the Netherlands, the Nijmegen Gender Awareness in Medicine Scale (N-GAMS). The aim of this study was twofold: 1. to measure gender awareness among students at the University of Lausanne and to assess the evolution of gender awareness throughout medical education; and 2. to validate the N-GAMS scale in a French speaking setting.

## Methods

### Study design and gender awareness measure

We performed an observational cross-sectional study among medical students at the medical school of the University of Lausanne in Switzerland. To measure student’s gender sensitivity we used the N-GAMS scale, which has been developed in 2008 and validated by the Dutch team of Radboud University Nijmegen Medical Centre [7]. This scale is based on two attitudinal aspects of gender-awareness: gender sensitivity (GS) and gender role ideology which is assessed towards patients (GRIP) or doctors (GRID). The three subscales contain statements that students have to assess using a 5 Likert point scale (ranging from 1 “not agree at all” to 5 “totally agree”). Some statements have reverse meaning, therefore an adjustment of reverse scoring statements was done. The GS group has 14 statements, which explore the student’s general opinion of considering gender and sex in healthcare, for example with statements such as the following “Physicians’ knowledge of gender differences in illness and health increases quality of care”. The GRIP score has 11 statements which specifically relate to stereotypes about male or female patients and their communication regarding health problems, with statements such as: “Women expect too much emotional support from physicians”. The GRID score has 7 statements, which explore student’s stereotypes towards doctors and their practice, for example: “Male physicians are more efficient than female physicians”. A higher score in the GS statements means a higher gender sensibility. On the GRIP and GRID scales high score implies more gender-stereotyping opinions. It is to our knowledge the only validated scale that measures gender awareness in medical students. It has been developed and validated in the Netherlands and used in two other studies in Sweden [20] and in Taïwan [21].

A professional interpreter translated the N-GAMS scale from Dutch to French. The French questionnaire was then tested by three medical students and two members of the study team (IR and CC) and adapted according to their comments. Translation of the English questionnaire was also done by the study team into French and then back translated into English. Results were then compared with the translation done by the professional interpreter and disagreements discussed and resolved. We offered the possibility for students to add comments at the end of the survey, to obtain a qualitative opinion about the questionnaire. Additional file 1 shows the statements of the N-GAMS questionnaire translated in French. An English version of the N-GAMS questionnaire can be found in *Andersson* et al. (with minor modification) [20]*,* and the initial version in English in *Verdonk* et al. [7] (https://bmcmededuc.biomedcentral.com/articles/10.1186/1472-6920-12-3/tables/1).

### Study population

The survey instrument - a questionnaire containing the N-GAMS scale as well as basic demographic data – was sent to all medical students of UniL during the academic year of 2016–2017, using an anonymous online survey (Surveygizmo® software). In total 1686 registered students were invited to participate, with a majority of women (62.6% female students). About 40% of registered students were first year medical students and the number decreases and stabilizes after the 2nd year of medical school. Table 1 shows the proportion of students in each academic year and the proportion of female students registered for each year. The recruitment of participants was conducted through e-mails. Announcements in various Facebook students’ groups were also posted. The survey was initially open for a month. As we noticed a lack of male participants, we encouraged their participation through a second targeted e-mails.

**Table 1:** Number of participants stratified by gender and years of education, with total number of students registered in Autumn 2016 in the University of Lausanne in parentheses

Year of study	Number of male participants (male students in total)	Number of female participants (female students in total)	Total number of participants (total number of students)	Participation rate (%)	Percentage of female students in total (%)
1	20 (200)	74 (468)	95^a^(668)	14.20%	70%
2	29 (103)	43 (135)	72 (238)	30.20%	56.70%
3	27 (84)	35 (110)	62 (194)	31.90%	56.70%
4	23 (82)	33 (122)	56 (204)	27.40%	59.80%
5	39 (90)	28 (95)	67 (185)	36.20%	51.40%
6	12(68)	32 (121)	44 (189)	23.20%	64%
Total	150 (627)	245 (1051)	396 (1678)	23.60%	62.60%

^a^ Category “other” not included

### Statistical analysis

Exploratory factor analysis was used in order to define N-GAMS subscales. At first impression, it appeared that 2 factors were enough, one for gender sensitivity and a second for gender stereotypes (GRIP and GRID). Following previous work (Verdonk, 2008; Andersson, 2012), we “forced” 3 factors. In order to have three separate factors, scores with loading smaller than a cut-off of 0.4 and *cross-loading* scores (scores with loadings > 0.4 on more than one factor) were dropped leading to define three relevant dimensions. Reliability of the three subscales above was assessed calculating the alpha Cronbach coefficient. Mean subscales were calculated for male and female students and compared using two sample t-tests. A linear model was built with each subscale as a dependent variable and students’ sex and age as covariables. Quadratic effect of age and interaction between age and sex were tested.

## Results

In total, 560 students answered the survey (33% of registered students), with 396 students who completed the questionnaire, resulting in a final response rate of 23.4%. The proportion of participants varied between 14.2% (1st academic year) to 36.2% (5th academic year). Among included students, 245 were women (61.9%), 150 men (37.9%) and one participant was categorized as “other” and excluded from analyses. There were more female participants in every year, except for the 5th academic year, where 58.2% of respondents were men. Age of students ranged from 18 to 32 years old, with a mean of 22 years old*.* Age corresponds to year of study but was preferred because of the low number of samples for each year. Table 1 shows the participation rate of students for each academic year.

With an exploratory factor analysis conducted on 374 completed questionnaire (21 responses were excluded that had one or several missing values on N-GAMS scores), we obtained 3 relevant subscales, GS, GRIP and GRID, globally explaining 40% of data variability. The first subscale represented gender sensitivity (GS; the higher the score value, the higher the sensibility to gender issues) and was defined by the mean of 10 out of the 14 original statements. Eight of them were reversed since they presented a negative loading in the factor analysis. The second subscale represented stereotypes towards patients (GRIP: the higher the score value the stronger the stereotypes) and was defined by the mean of 9 out of the 11 original statements. The third subscale represented stereotypes towards doctors, (GRID: the higher the score value the stronger the stereotypes) and was defined by the mean of 4 out of the 7 original statements. Reliability scores of the N-GAMS subscales measured by Cronbach’s alpha were α = 0.79 for the GS subscale, α = 0.88 for the GRIP subscale and 0.77 for the GRID subscale. Therefore N-GAMS could be validated with 3 relevant subscales (see Additional file 2).

**Fig. 1:**
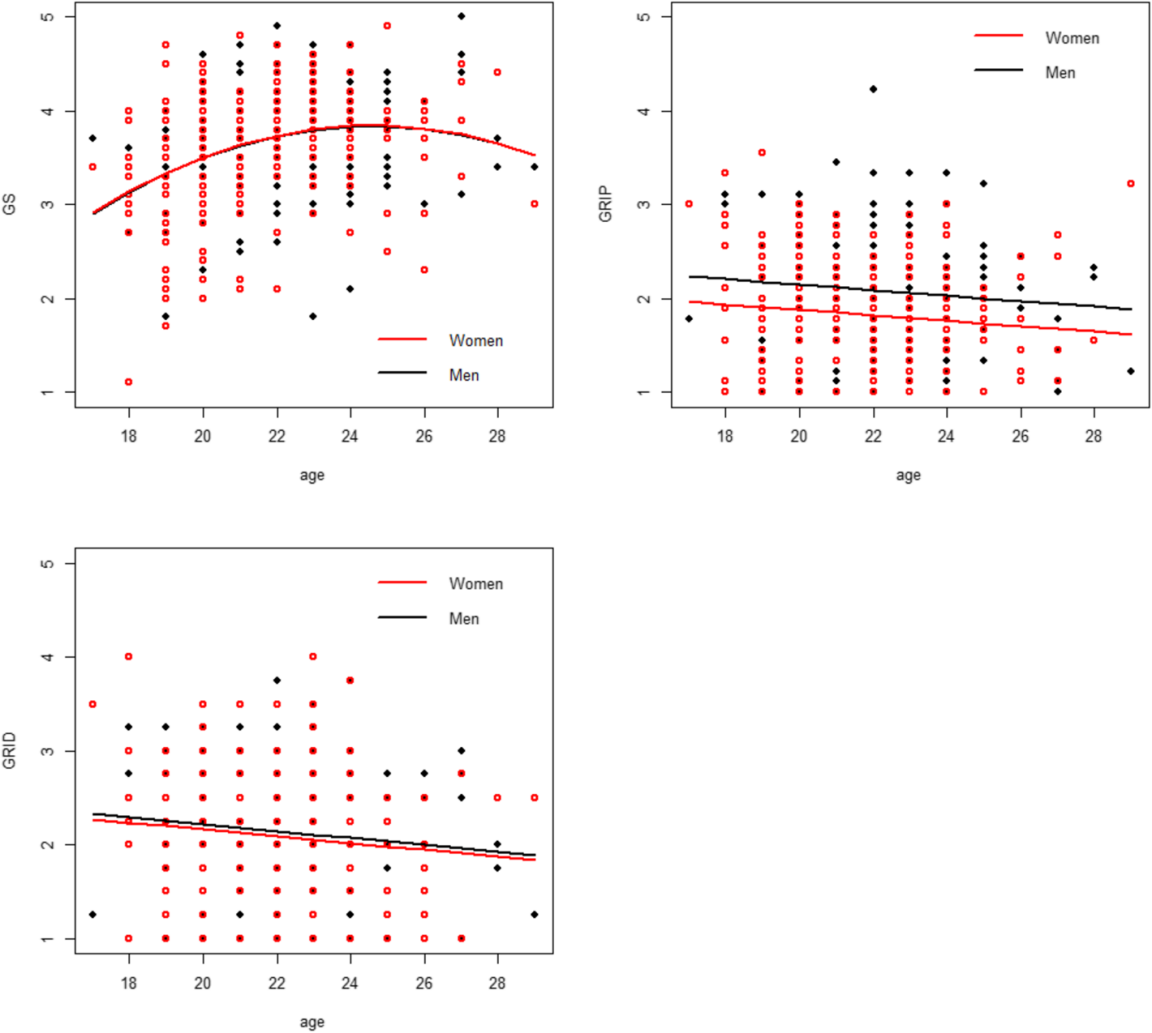
Predicted mean subscales according to the linear model, with student’s age and sex

The students scored a GS subscore of 3.65 (SD 0.63), a GRIP subscore of 1.92 (SD 0.62) and a GRID subscore of 2.11 (SD 0.71). As shown in Table 2, GS and GRID subscores were not significantly different between female and male students (GS 3.62 for women, 3.70 for men, *p* = 0.27, GRID 2.10 for women, 2.13 for men, *p* = 0.76). A significant difference was found with the GRIP subscale, with a mean score of 1.83 for women and 2.07 for men (*p* < 0.001), which suggests a more stereotyped opinion toward patients among male students. A trend was observed with age (Table 3*and* Fig. 1): gender sensitivity showed a significant quadratic trend with age, with an initial increase followed by a stabilization (both linear and quadratic effect p < 0.001); stereotypes towards patients and doctors decreased linearly with students getting older (GRIP *p* = 0.04, GRID *p* = 0.02). Adjusting for age, students’ sex was still associated with GRIP subscale, female students having less tendency to have stereotyped beliefs (Table 3; coefficient 0.27, *p*-value < 0.001).

**Table 2:** Mean subscores stratified by sex

	Total	Women	Men	p of the difference
GS score	3.65 (0.63)	3.62 (0.63)	3.70 (0.63)	0.270
GRIP score	1.92 (0.62)	1.83 (0.56)	2.07 (0.67)	< 0.001
GRID score	2.11 (0.71)	2.10 (0.70)	2.13 (0.73)	0.758

Response varies from 1 = not agree at all to 5 = totally agree

GS Gender sensitivity, GRIP Gender Role Ideology towards Patients, GRID

Gender Role Ideology towards Doctors

**SD in parentheses **

**Table 3:** Linear model with students’ sex and age

	Student’s sex (Men vs Women)	Age	Age2
GS score	0.005 (0.942)	0.80 (< 0.001)	− 0.02(< 0.001)
GRIP score	0.27(< 0.001)	−0.03 (0.035)	**–**
GRID score	0.06 (0.472)	−0.04 (0.024)	**–**

Response varies from 1 = not agree at all to 5 = totally agree

GS Gender sensitivity, GRIP Gender Role Ideology towards Patients, GRID Gender Role Ideology towards Doctors

***p*in parentheses**

We collected 36 qualitative comments at the end of the N-GAMS questionnaire. Most participants complained about the formulation of the statements, which were too stereotypical and suggesting negative stereotypes towards women. Some of them suggested adding also negative stereotyped statements about men to balance the questionnaire, which addressed negative roles for women only.

## Discussion

Using the N-GAMS instrument, we obtained a general overview of gender awareness of medical students from Lausanne’s Medical School and identified some patterns. Students had overall medium to high gender sensitivity and medium to low gender stereotypes. Women had significantly less stereotypes toward patients than men. Gender sensitivity and gender stereotypes toward doctors were not significantly different between male and female students. We observed both a positive improvement of gender sensitivity and a decrease in gender stereotypes toward patients and doctors over the years, suggesting an improvement of gender awareness when students move forward in their medical curriculum.

The finding that female students had less stereotypes towards patients may be partially explained by the fact that women in general are more aware of stereotypes toward patients because it speaks about their own position and their right to a better health care. The high GRIP score, showing stereotypes toward patients, among 3rd and 4th year male students can be associated with the absence of gender-focused lectures during the first clinical master's years. The improvement of gender awareness during the master’s years might also be explained by the start of the clinical years in the 3rd year, when students are confronted with patients and clinical situations putting their knowledge into practice. During this process, most of the diseases are described with scores and guidelines based on clinical research and prevalence, which contain often a gender or sex aspect. Students start to sort out diseases influenced by gender and sex patterns and develop stereotypes. This could be prevented by an implemented gender dimension in all lectures including specialties, where the role and influence of gender is addressed. An improvement of general gender awareness could be also achieved by implementing gender focused courses also at a clinical level, for nursing staff, senior clinicians or attended specialists, who are usually responsible for medical students in the first years of clinical learning.

We compared our results with other countries based on the studies published in the Netherlands and in Sweden in 2012 [20]*.* Results suggest a better gender sensitivity of Swiss students as shown by higher mean GS score (GS score 3.62 for women and 3.70 for men), when compared to Swedish students (GS score 3.37 for women and 3.30 for men) and to Dutch students (GS score 3.43 for both sexes). Swiss male students had more stereotypes towards patients than Swedish male students (GRIP 2.07 in Swiss compared to 1.96 in Swedish students) but had less stereotypes towards doctors than Dutch male students (GRID 2.13 in Swiss compared to 2.44 in Dutch students). Another aspect is the influence of sociocultural norms, including gender norms that differ across countries. The social status of women is stronger in Sweden, where gender equality is ensured in more dimensions than in Switzerland. For example, according to Swedish Statistics, 18% of employed women have part-time jobs and the gender gap in salaries was 12% in 2017 [22]. The gender gap in Netherlands was 21% in the same year [23]. Sweden is ranked first in the EU to have the most equitable sharing of households activities [23]. The comparison is also observed in the international World Economic Forum Global Gender Gap Index (http://reports.weforum.org/global-gender-gap-report-2016/rankings/), where Sweden ranks 4th, Netherland 16th and Switzerland 11th. We suggest that these differences of women’s social status can explain the differences in gender awareness across countries. In addition, the time lag between studies (the Andersson study was performed in 2006–2009 and our study in 2017) may explain the discrepancies in gender awareness between students. Over time the public opinion about gender awareness in general has evolved. With the #metoo era and the feminist wave increasing in social media, considering the timeline is important [24]. The social environment, including in the work, scientific and medical sectors, is changing and gender inequalities has become a prominent topic including in the media. Those factors might explain a higher gender sensitivity in the participants in our study. Comparison were not possible with the study from Taiwan, because the N-GAMS scores had been modified.

The differences between educational systems play an important role in the results. In Lausanne, in the first year the students are approached by a gender focused lecture of one hour and an optional seminar which depicts the culture of the faculty about the importance of gender. In Sweden and the Netherlands at the time of study, the implementation of gender aspect in the medical curricula was already in place [20]. The educational background in the universities influenced the differences in scores.

Comparing our study with the Swedish and the Dutch studies has its limitation due to the difference in the educational level of the participants. The data was collected from first year medical students in the Andersson study, whereas in our study all six academical years could participate. In 2006–2007 the participation rate was 94% for Netherlands and 93% for Sweden [20]. Their sample sizes were greater than in our study, which limits comparisons.

### Limitations

Our study has some limitations that have to be acknowledged. First, the N-GAMS questionnaire has some pitfalls. The instrument is based on formulated negative stereotypes to which participants are asked to react and the formulation of such stereotypes is context and time-bond. Hence, a linear translation of these formulations may not always be adequate. In addition, the use of negative stereotypes may have induced a social desirability response bias. Finally, a back-translation of the questionnaire from Dutch to French was not formally made. However, we did translate the English questionnaire into French and then back into English, confronting this version with the one ot the professional interpreter. We thus aimed to limit the risk of misinterpretation.

The participation rate was low (23.4%) and 8% of the students answered but did not complete the questionnaire and were excluded from analyses. We thus cannot exclude a selection bias. Indeed, male students were underrepresented and had to be encouraged by a second reminder to answer the survey which showed success as the sample sex ratio matched the real population ratio. First year medical students were overrepresented and, even if the participation rate was proportional to the total number students, they might have influenced the overall results, because they did not have gender courses at the time of the survey. Due to our small sample, we were not able to stratify results by medical year to look at the influence of existing gender medicine courses in the reduction of gender bias. It is possible that students sensitized or interested by the gender dimension in health answered the survey in a larger proportion and were thus over-represented. If this holds true, it means that we might expect a lower gender awareness compared to what we have measured. The N-GAMS instrument is to our knowledge the only validated questionnaire that exists to measure gender awareness. It has been criticized [20] and might not be sensitive enough to fully reflect gender awareness; it has allowed obtaining a general overview of Swiss students’ gender awareness, but did not allow a more precise understanding of which kind of stereotypes were in play.

### Strengths

We used a validated tool (N-GAMS) to specifically assess gender awareness among medical students. This study permitted the validation of N-GAMS in French and validated its utilization in Switzerland, which will allow its application in other French-speaking countries. By adding a comments section in the questionnaire, we gave the students the opportunity to assess the statements of N-GAMS and give qualitative insights on the questionnaire and express their opinion. Finally, our study is the first, to our knowledge, to have assessed gender awareness among Swiss medical students and will serve as a baseline for further comparison with other countries or within the same setting, to assess the impact of a better inclusion of the gender dimension in medical education.

## Conclusion

Through their participation in this study, medical students at the University of Lausanne showed a certain interest in the topic of gender in medicine but appear to have stereotypes and suboptimal gender sensitivity as shown by our results. The evolution of gender awareness throughout the academic years shows promising results but implementing coordinated and continuous teaching of the gender dimension throughout the whole medical curriculum is necessary to prevent stereotypes and bias affecting future doctors, and ultimately future patients. In addition, despite some weaknesses, the N-GAMS instrument could be adapted to different countries and languages. An early sensitization on gender bias and their influence on health among medical students in Swiss Universities could contribute to improve the quality of medical care and ensure equity in healthcare.

## Supplementary information

**Additional file 1.** Nijmegen Gender Awareness in Medicine Scale (N-GAMS).

**Additional file 2.** Results of factor analysis with three factors. First three graphs represent plots of pairs of factors (GRIP,GS); (GRID,GS), and (GRID,GRIP). All scores have large (>0.4) loading on one (and only one) factor. The last graph gives 4 methods for choosing the number of factor retained. 2 on 4 methods give 3 factors (ones dropped cross-loading scores, otherwise all methods give only two factors.).

## Data Availability

Our data are not on a data repository. The datasets used and/or analysed during the current study are available from the corresponding author on reasonable request. Only coded data may be shared.

## Abbreviations

N-GAMS: Nijmegen Gender Awareness Medical Scale
UniL: University of Lausanne
GS: Gender Sensitivity
GRIP: Gender Role Ideology towards Patients
GRID: Gender Role Ideology towards Doctors
PlaGe: Plateforme en études genre
OFS: Office fédéral de la statistique

## References

[1] Doyal L (2001). Sex, gender, and health: the need for a new approach. BMJ (Clinical research ed).

[2] Ruiz-Cantero MT, Vives-Cases C, Artazcoz L, Delgado A, Calvente MDMG, Miqueo C, et al. A framework to analyse gender bias in epidemiological research. J Epidemiol Community Health. 2007;61(SUPPL. 2). 10.1136/jech.2007.062034 PMC2465769 18000118

[3] Risberg G, Johansson EE, Hamberg K (2009). A theoretical model for analysing gender bias in medicine. Int J Equity Health.

[4] Equality EI for G. “Gender awareness”. Gender Equality Glossary and Thesaurus Available from: https://eige.europa.eu/taxonomy/term/1147. Accessed 9 July 2019.

[5] Verdonk P, Benschop YWM, De Haes HCJM, Lagro-Janssen TLM (2009). From gender bias to gender awareness in medical education. Adv Health Sci Educ.

[6] Hamberg K (2008). Gender bias in medicine. Womens Health (London, England).

[7] Verdonk P, Benschop YWM, De Haes HCJM, Lagro-Janssen TLM (2008). Medical Students’ Gender Awareness. Sex Roles.

[8] Regitz-Zagrosek V, Oertelt-Prigione S, Prescott E, Franconi F, Gerdts E, Foryst-Ludwig A (2016). Gender in cardiovascular diseases: impact on clinical manifestations, management, and outcomes. Eur Heart J.

[9] Liaudat CC, Vaucher P, De FT, Jaunin-Stalder N, Herzig L, Verdon F (2018). Sex/gender bias in the management of chest pain in ambulatory care. Womens Health.

[10] Regitz-Zagrosek V (2017). Gender and cardiovascular diseases : why we need gender medicine. Internist.

[11] Verdonk P, Benschop Y, de Haes H, Mans L, Lagro-Janssen T (2009). “Should you turn this into a complete gender matter?” gender mainstreaming in medical education. Gend Educ.

[12] OFS. Egalité entre femmes et hommes: la Suisse en comparaison internationale. Neuchâtel; 2008.

[13] OFS. Le travail à temps partiel en Suisse 2017 [Internet]. Neuchâtel; 2019. Available from: https://www.bfs.admin.ch/bfs/fr/home/actualites/quoi-deneuf.assetdetail.7106888.html. Accessed 9 July 2019.

[14] Inégalités salariales: les femmes ont gagné 19,6% de moins que les hommes en 2016 [Internet]. Neuchâtel; 2019. Available from: https://www.bfs.admin.ch/bfs/fr/home/statistiques/catalogues-banques-donnees/communiques-presse.assetdetail.7206414.html. Accessed 5 Nov 2019.

[15] Begert L. Egalité des genres dans la profession médicale : la Suisse, un pays pionnier ? Survol historique et sociologique (1865–2015) {Unpublished doctoral thesis}. Institut des Humanités en Médecine : Université de Lausanne; 2019.

[16] Pro-Femmes C. Plan d’action AGIR+ pour l’égalité 2017–2020 de la Faculté de biologie et de médecine de l’Université de Lausanne dans le cadre de la poursuite du projet « Vision 50/50 » de la Direction de l’UNIL [Internet]. Lausanne; 2017. Available from: https://www.unil.ch/fbm/home/menuinst/la-releve-academique/egalite-femmes-hommes/plan-daction-de-la-fbm.html. Accessed 5 Nov 2019.

[17] Fussinger C. Intégrer le genre dans la formation médicale prégraduée: peut-on transférer l’expérience néerlandaise sur sol suisse?: étude exploratoire menée au sein de l’École de médecine de l’Université de Lausanne (FBM/UNIL) pour l’année académique 2009–2010. Département universitaire de médecine et de santé communautaires; 2011.

[18] UNIL (2014). Plateforme interfacultaire en études genre-Rapport d’activité 2013-2014.

[19] Clair C, Schwarz J. https://www.unil.ch/ecoledemedecine/fr/home/menuguid/enseignante/medecine-et-genre.html. Accessed 9 July 2019.

[20] Andersson J, Verdonk P, Johansson EE, Lagro-Janssen T, Hamberg K (2012). Comparing gender awareness in Dutch and Swedish first-year medical students - results from a questionaire. BMC Med Educ.

[21] Chung YC, Lin CY, Huang CN, Yang JH (2013). Perceptions on gender awareness and considerations in career choices of medical students in a medical school in Taiwan. Kaohsiung J Med Sci.

[22] Statistics Sweden. Women and Men in Sweden 2016. 2016. Available from: https://www.scb.se/contentassets/4550eaae793b46309da2aad796972cca/le0201_2017b18_br_x10br1801eng.pdf. Accessed 10 Apr 2019.

[23] European Institute of Gender Equality. Gender Equality Index 2017: Netherlands, vol. 1; 2017. p. 1–6. Available from: https://eige.europa.eu/rdc/eige-publications/gender-equality-index-2017-denmark. Accessed 10 Apr 2019.

[24] Neil AO, Sojo V, Fileborn B, Scovelle AJ, Milner A (2018). The # MeToo movement: an opportunity in public health?. The Lancet.

