# Supplementary material for: Gender awareness among medical students in a Swiss University
Source: BMC Med Educ. 2020 Jun 3;20:156. doi: 10.1186/s12909-020-02037-0 (PMC7268694; doi:10.1186/s12909-020-02037-0)
Supplement: Supplementary file 1 — Additional file 1. Nijmegen Gender Awareness in Medicine Scale (N-GAMS). [file 12909_2020_2037_MOESM1_ESM.pdf]

Additional file 1.

Nijmegen Gender Awareness in Medicine Scale (N-GAMS)

Translated from the dutch version

GS : Gender sensitivity

GS1 Tenir compte des différences entre homme et femme engendre l'inégalité dans la prise en charge

GS2 La connaissance des différences de genre homme/femme en bonne santé et en situation de maladie améliore pour les médecins la qualité de prise en charge

GS3 Les médecins doivent tenir compte uniquement des différences biologiques entre hommes et femmes

GS4 En ce qui concerne les affections non spécifiques au sexe, le sexe et/ou le genre du patient n'a aucune importance

GS5 Un médecin doit s'en tenir autant que possible aux aspects médicaux des symptômes des hommes et des femmes

GS6 Les médecins n'ont pas besoin de connaître quoi que ce soit de la vie personnelle des hommes et des femmes pour dispenser des soins médicaux

GS7 Les différences entre médecins hommes et médecins femmes sont trop faibles pour avoir de l'importance

GS8 C'est précisément parce que les hommes et les femmes sont différents que les médecins doivent traiter tout le monde de la même façon

GS9 Les médecins qui tiennent compte des différences de genre ne s'occupent pas des problèmes importants

GS10 Dans le cadre de la communication avec les patients, le fait que le patient soit un homme ou une femme n'a aucune importance pour un médecin

GS11 Dans le cadre de la communication avec les patients, peu importe que le médecin traitant soit un homme ou femme

GS12 Les différences entre les patients de sexe masculin et les patients de sexe féminin sont tellement faibles que les médecins peuvent difficilement en tenir compte

GS13 Pour un traitement efficace, les médecins doivent tenir compte des différences de genre en ce qui concerne le déroulement et les conséquences de la maladie

GS14 Il n'est pas nécessaire de considérer les différences de genre des patients lors de la description de leurs plaintes

GRIP : Gender role ideology toward patients

GRIP1	Les patients de sexe masculin comprennent mieux la façon de travailler des médecins que les patients de sexe féminin
GRIP2	Les patients de sexe féminin ont des attentes déraisonnables de la part des médecins en comparaison avec les patients de sexe masculin
GRIP3	Les femmes ont plus tendance que les hommes à aborder des thèmes avec le médecin qui n’ont pas leur place dans un cabinet de consultation
GRIP4	Les femmes attendent plus de soutien affectif que les hommes de la part des médecins
GRIP5	Les patients de sexe masculin sont moins exigeants que les patientes de sexe féminin
GRIP6	Les femmes ont plus recours aux services de santé que nécessaire
GRIP7	Les hommes ne vont pas consulter un médecin pour des problèmes de santé mineurs
GRIP8	Des affections médicalement inexplicables se manifestent chez les femmes parce qu’elles sont trop préoccupées par leur santé
GRIP9	Les patientes de sexe féminin se plaignent de leur santé parce qu’elles exigent plus d’attention que les patients de sexe masculin
GRIP10	Il est plus facile de déceler la cause des symptômes chez les hommes parce qu’ils disent directement de quoi il s’agit
GRIP11	Les hommes font plus souvent appel aux services de santé pour des problèmes qu’ils auraient pu prévenir

GRID : Gender role ideology toward doctors

GRID1	Les médecins de sexe masculin attachent trop d’importance aux aspects techniques de la médecine en comparaison avec les médecins de sexe féminin
GRID2	Les médecins de sexe féminin consacrent beaucoup trop de temps à leur consultation en comparaison avec les médecins de sexe masculin
GRID3	Les médecins de sexe masculin sont plus efficaces que les médecins de sexe féminin
GRID4	Les médecins de sexe féminin ont plus d’empathie que les médecins de sexe masculin
GRID5	Les médecins de sexe féminin tiennent beaucoup trop compte de la vie personnelle du patient
GRID6	Les médecins de sexe masculin sont plus capables d’assumer leur travail que les médecins de sexe féminin
GRID7	Les médecins de sexe féminin sont trop impliquées émotionnellement avec leurs patients
