# Supplementary material for: Gender awareness among medical students in a Swiss University
Source: BMC Med Educ. 2020 Jun 3;20:156. doi: 10.1186/s12909-020-02037-0 (PMC7268694; doi:10.1186/s12909-020-02037-0)

Additional file 2.

Results of factor analysis with three factors. First three graphs represent plots of pairs of factors (GRIP,GS); (GRID,GS), and (GRID,GRIP). All scores have large (>0.4) loading on one (and only one) factor. The last graph gives 4 methods for choosing the number of factor retained. 2 on 4 methods give 3 factors (ones dropped cross-loading scores, otherwise all methods give only two factors.)

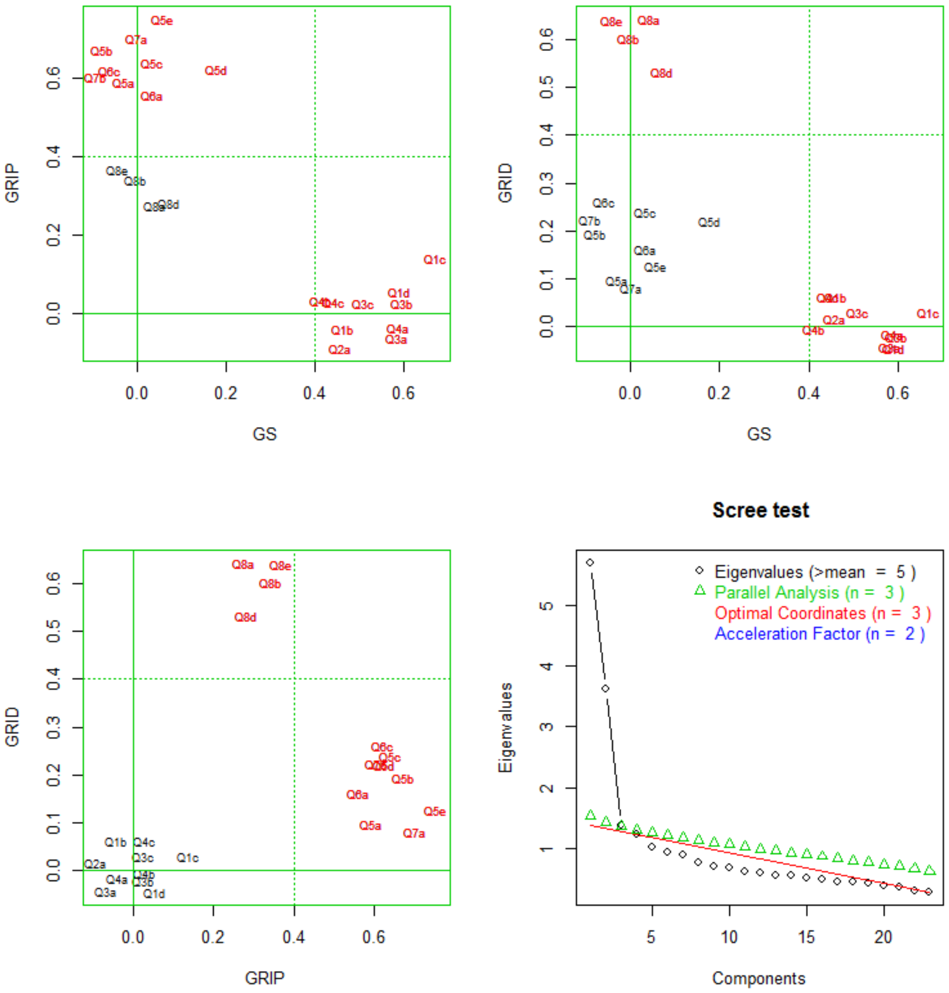

Supplement: Supplementary file 2 — Additional file 2. Results of factor analysis with three factors. First three graphs represent plots of pairs of factors (GRIP,GS); (GRID,GS), and (GRID,GRIP). All scores have large (>0.4) loading on one (and only one) factor. The last graph gives 4 methods for choosing the number of factor retained. 2 on 4 methods give 3 factors (ones dropped cross-loading scores, otherwise all methods give only two factors.). [file 12909_2020_2037_MOESM2_ESM.pdf]
